# Prognostic value of radiomics-based hyperdense middle cerebral artery sign for patients with acute ischemic stroke after thrombectomy strategy

**DOI:** 10.3389/fneur.2022.1037204

**Published:** 2023-01-13

**Authors:** Linna Li, Mingyang Li, Zhongping Chen, Fei Lu, Min Zhao, Huimao Zhang, Dan Tong

**Affiliations:** ^1^Department of Radiology, The First Hospital of Jilin University, Changchun, Jilin, China; ^2^Pharmaceutical Diagnostics, GE Healthcare, Beijing, China

**Keywords:** hyperdense middle cerebral artery, acute ischemic stroke, mechanical thrombectomy, prognostic factor, radiomics

## Abstract

**Background and purpose:**

The purpose of this study was to evaluate the prognostic value of radiomics-based hyperdense middle cerebral artery sign (HMCAS) for patients with acute ischemic stroke (AIS) after mechanical thrombectomy (MT) and to establish prediction models to identify patients who may benefit more from MT.

**Methods:**

In this retrospective study, a total of 102 consecutive patients who presented with HMCAS on non-contrast computed tomography (NCCT) at admission and underwent MT in our hospital between January 2019 and December 2020 were recruited. Among them, 46 experienced favorable outcomes (modified Rankin Scale [mRS] ≤ 2) at 3 months of follow-up. All patients were categorized into two sets, namely, the training set (*n* = 81) and the test set (*n* = 21). Radiomics features (RFs) and clinical features (CFs) in the training set were selected by statistical analysis to create models. The models' discriminative ability was quantified using the area under the curve (AUC) and confirmed by decision curve analyses.

**Results:**

The prediction model established using CFs before MT includes baseline National Institutes of Health Stroke Scale (NIHSS) and neutrophil-to-lymphocyte ratio (NLR) [AUC [95% confidence interval (CI)] = 0.596 (0.312–0.881)]. A total of 1,389 RFs were extracted from each hyperdense territory and 8 RFs were left to build the radiomics model [RM; AUC (95%CI) = 0.798 (0.598–0.998)]. The model using preoperative CFs and RFs showed good performance [AUC (95%CI) = 0.817 (0.625–1.000)]. The models using post-operative CFs alone [AUC (95%CI) = 0.856 (0.685–1.000)] or post-operative CFs with RFs [AUC (95%CI) = 0.894 (0.757–1.000)] also showed good discrimination.

**Conclusion:**

The radiomics-based HMCAS might be a promising tool to predict the prognoses of patients with AIS after MT.

## Introduction

Non-contrast computed tomography (NCCT) is currently the most available diagnostic imaging modality that can identify early signs of ischemic stroke and hemorrhage. NCCT scan at admission reveals not only early ischemic changes in the brain parenchyma but also hyperdense artery sign (HAS) in patients with emergent large vessel occlusion (LVO) (1, 2). HAS is most commonly found in the middle cerebral artery (MCA) on initial NCCT for patients with acute ischemic stroke (AIS) known as the hyperdense middle cerebral artery sign (HMCAS), indicating acute MCA and/or terminal internal carotid artery (ICA) occlusion with high red blood cell aggregation (1, 3, 4). HMCAS was considered to be associated with increased stroke severity, large infarct volume, and poor functional outcome in the pre-thrombectomy era (5). However, the prognostic value of HMCAS for predicting the outcomes of patients undergoing mechanical thrombectomy (MT) remains inconclusive.

Radiomics analysis is an emerging approach that converts imaging data into a high-dimensional feature space using automatically extracted data-characterization algorithms (6). Several studies have reported that clot-based radiomics is predictive of treatment response after MT strategy for AIS (7–10). This suggests that the radiomics features (RFs) extracted from pretherapeutic radiological imaging may provide valuable information about the clot composition and structure, which may influence the prognosis. In this study, we hypothesized that radiomics-based HMCAS on NCCT could provide more prognostic information for patients with AIS after MT. Therefore, the predictive ability of radiomics-based models, with or without clinical features (CFs) before and after MT, was evaluated in our study.

## Materials and methods

### Patients

**Figure 1:**
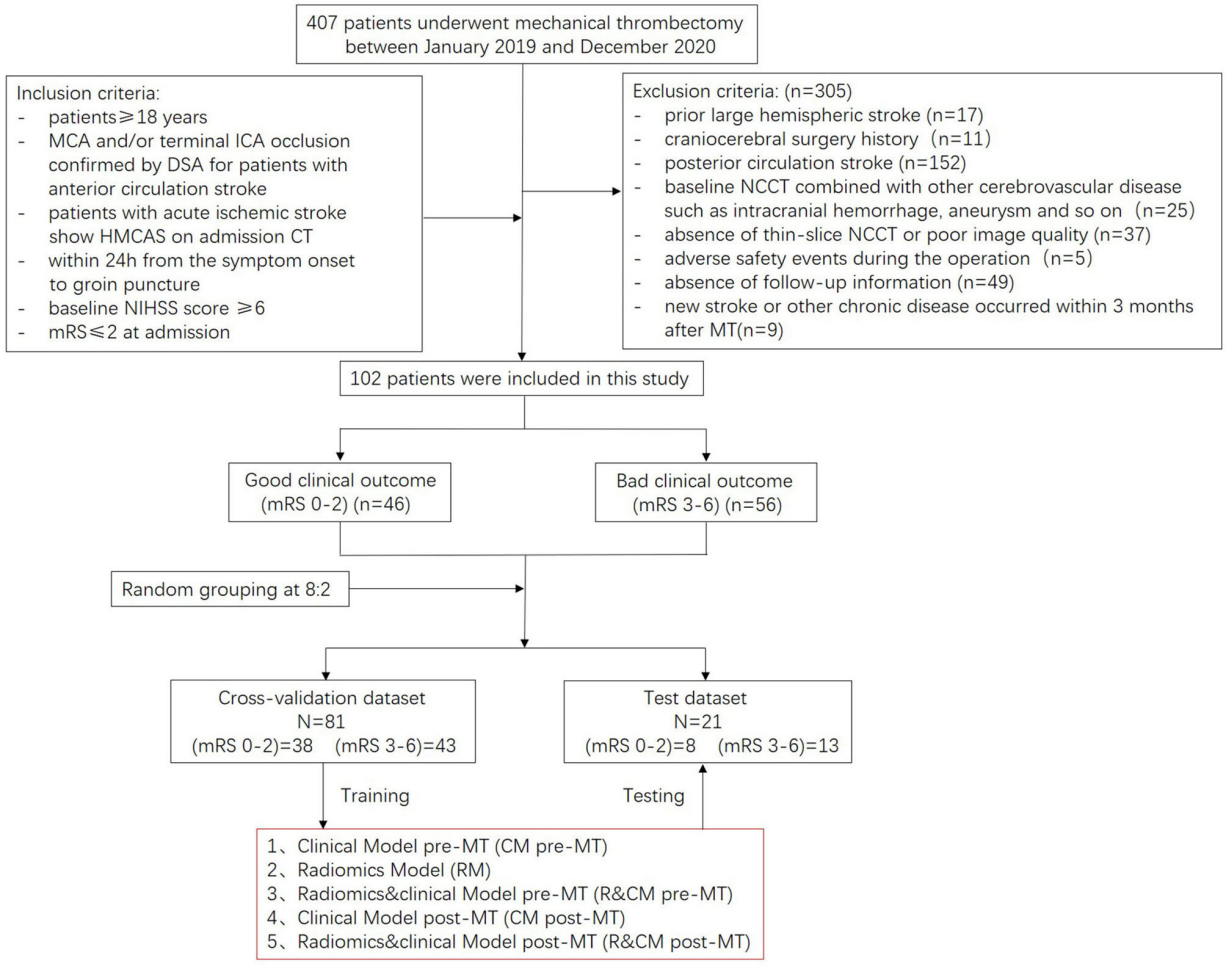
The recruitment process.

**Figure 2:**
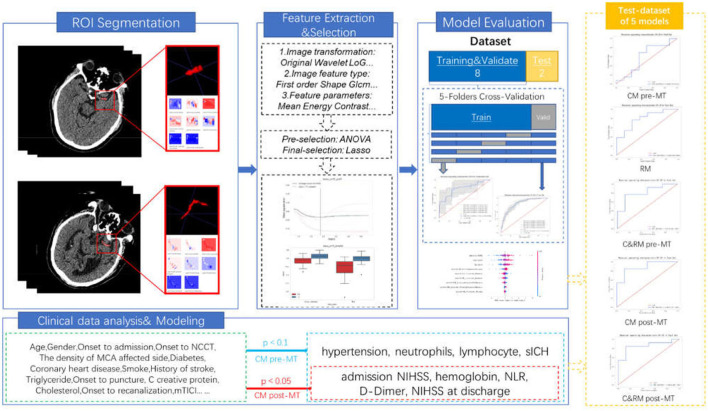
The workflow of this study.

In this retrospective study, the NCCT imaging and clinical data of 407 patients who were diagnosed with AIS caused by LVO and underwent MT in our hospital between January 2019 and December 2020 were screened. The inclusion criteria were as follows: (1) patients ≥ 18 years, (2) the diagnosis of MCA and/or terminal ICA occlusion was confirmed by digital subtraction angiography (DSA) for patients with anterior circulation stroke, (3) patients were diagnosed with AIS and presented with HMCAS on admission NCCT, (4) within 24 h from the symptom onset to groin puncture, (5) the conditions of patients were evaluated with baseline NIHSS score ≥6, and (6) modified Rankin Scale (mRS) ≤ 2 at admission. Patients with prior large hemispheric stroke (*n* = 17), craniocerebral surgery history (*n* = 11), posterior circulation stroke (*n* = 152), baseline NCCT combined with other cerebrovascular diseases such as intracranial hemorrhage, aneurysm, and so on (*n* = 25), absence of thin-slice NCCT or poor image quality (*n* = 37), adverse safety events during the operation (*n* = 5), absence of follow-up information (*n* = 49), and new stroke or other chronic diseases occurred within 3 months after MT (*n* = 9) were excluded. Finally, 102 patients were included in this study. The recruitment process of the study is shown in Figure 1, and the workflow of the study is shown in Figure 2.

### CFs collection

The following CFs of all patients were collected: (1) baseline information, including age, sex, medical history of hypertension, diabetes mellitus, atrial fibrillation, coronary heart disease, stroke, and history of smoking and drinking; (2) laboratory examination results, including the levels of hemoglobin, white blood cells (WBCs), neutrophils, lymphocytes, platelets, NLR, C-reactive protein, fibrinogen, D-dimer, cholesterol, and triglyceride; and (3) other clinical information, including the time from onset to admission, from onset to NCCT examination, from onset to puncture, from a puncture to recanalization, and from onset to recanalization. The baseline NIHSS score was recorded by two experienced neurologists before endovascular treatment.

All operations are performed by doctors with more than 5 years of experience. The recanalization was considered successful when a modified Thrombolysis in Cerebral Infarction (mTICI) score of 2b/3 was documented by DSA, which was recorded during the operation and evaluated by experienced neurologists. Cranial CT was performed within 48 h after the operation to observe whether hemorrhage appeared (8). Symptomatic intracerebral hemorrhage (sICH) was described as hemorrhage accompanying a clinical worsening of ≥ 4 points on the NIHSS score (11). The stroke severity at discharge was assessed by the NIHSS. A good long-term clinical outcome was defined as an mRS score of 0–2 at 3 months after the operation, while a bad long-term clinical outcome was defined as an mRS score of 3–6.

### NCCT image acquisition and thrombus segmentation

Non-contrast computed tomography was performed using a 128-detector CT scanner (Brilliance iCT, Philips) with the following parameters: 120 kV, 79 mAs, section thickness of 1.0, 500 mm field-of-view, and 512 × 512 matrix. HMCAS was identified when the lumen of the MCA was denser than adjacent or equivalent contralateral arteries on NCCT but non-calcified. All HMCAS on NCCT images were visually evaluated by two trained neuroradiologists with 5 years of experience who were blinded to patients' baseline characteristics. A senior radiologist with 20 years of experience made the final decision in a case where there was disagreement. Simple imaging features including thrombus attenuation, contralateral MCA attenuation, and the length of clot were acquired by manual measurement before segmentation of ROIs and RFs extraction on axial NCCT images (12). Then, the difference and ratio of thrombus attenuation and contralateral MCA attenuation were calculated before statistical analysis.

The ROIs were drawn slice by slice from the axial view of NCCT images using ITK-SNAP (version 3.8.0, http://www.itksnap.org) by a junior and a senior neuroradiologist, as previous literature described (9). Plotting ROIs with a manual coating of thrombi revealed hyperdensity while viewing the corresponding DSA and/or computed tomographic angiography (CTA) images for guidance (Supplementary Figure 1). The RIAS software was used for image preprocessing, feature exaction, and model construction (13). To assess the repeatability of characteristic extraction between intra-observer and inter-observer, the RFs with intra-class correlation coefficients (ICCs) >0.9 were left. Then, a total of 1,389 RFs were extracted automatically from each ROI using Pyradiomics (version 3.0.0) (14). The ICC values of intra-observer and inter-observer reproducibility of simple imaging features were all >0.8 in Supplementary Table 2, showing a good agreement.

### Data standardization and gradient feature selection

**Figure 3:**
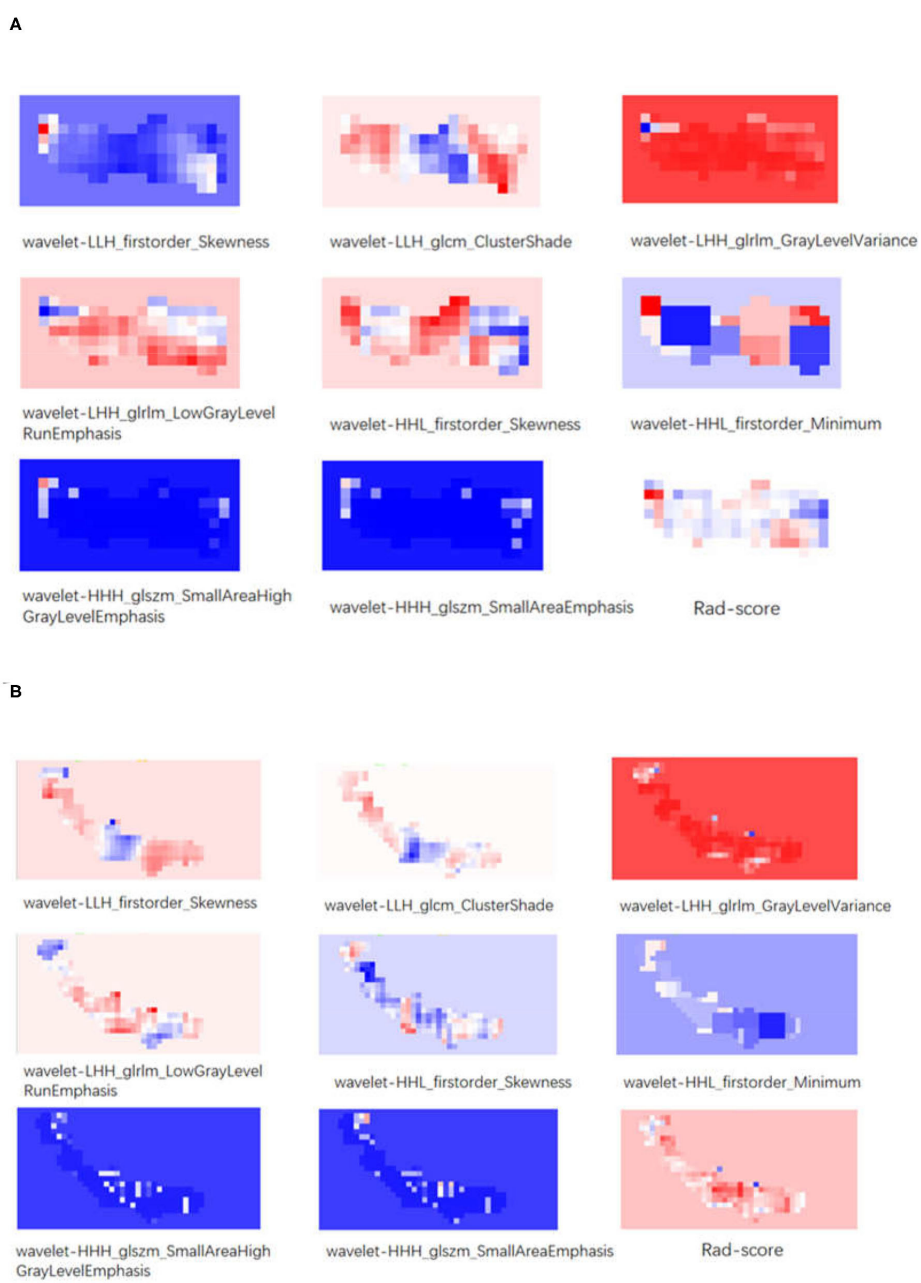
**(A)** The visualization of texture features and Rad-score of a patient with a good prognosis. **(B)** The visualization of texture features and Rad-score of a patient with a bad prognosis.

The total dataset was divided into a cross-validation set and a test set at a ratio of 8:2. Then, patients in the cross-validation set were further categorized into the training set and the validation set, which was used to perform 5-fold cross-validation and evaluate the comprehensive effectiveness of RFs. The test set is completely independent and applied to test the performance of the model. The mean and variance of the RFs were obtained from the cross-validation set and then used to standardize the test set. The gradient feature selection was used to evaluate 1,389 RFs of each NCCT image. Analysis of variance (ANOVA) was first used to select 12 RFs. The *p*-values of these features were all <0.05. Then, the 12 features were further selected by the least absolute shrinkage and selection operator (LASSO) algorithm with 10-fold cross-validation. The α-value was set as 0.0133 with the smallest mean square error, as shown in Supplementary Figure 2. Finally, 8 RFs were obtained. The examples of RF visualization of the two-group ROI images are shown in Figure 3.

### Statistical analysis

Statistical analysis was performed using the Python packages statsmodels (version 0.12.1, https://www.statsmodels.org/stable/index.html), Pingouin (version 0.5.2, https://pingouin-stats.org/index.html), and SciPy (version 1.5.3, https://scipy.org). Continuous variables were described as mean ± standard deviation or median with interquartile range, depending on the distribution of the variables. The normality of the data was assessed by the Kolmogorov–Smirnov and Shapiro–Wilk tests. MedCalc (version 20.121, https://www.medcalc.org) was used for the DeLong test of five modes.

Data were divided into two groups, namely, good prognosis (Label 0) and poor prognosis (Label 1). The chi^2^ test or Fisher's exact test was used to compare categorical variables. The Mann–Whitney *U*-test or independent *t*-test was used to compare continuous variables. ROC curves with a 95% confidence interval (CI) were plotted for each statistically significant variable to investigate the accuracy of prognosis prediction. A *p*-value of <0.05 was considered statistically significant. Some CFs with a *p*-value of <0.1 were used to establish the prediction model after comprehensive analysis.

### Establishment and comparison of prediction models

Five logistic regression models were developed to predict poor prognosis before and after MT based on RFs or/and CFs: (1) clinical model pre-MT (CM pre-MT) with preoperative CFs alone; (2) radiomics models (RMs) with RFs alone; (3) radiomic and clinical model pre-MT (R&CM pre-MT) with both RFs and preoperative CFs; (4) clinical model post-MT (CM post-MT) with post-operative CFs alone; and (5) radiomic and clinical model post-MT (R&CM post-MT) with both RFs and post-operative CFs. Then, the DeLong test was performed on the ROC curves of these models to assess possible overfitting.

### RFs-related score

Lasso-score is one of the fusion RFs analyzed by the LASSO algorithm. Another fusion RF (Rad-score) is acquired during model establishment.

Lasso-score and Rad-score were calculated as follows:

**Lasso-score** = wavelet-LLH_firstorder_Skewness ^*^ (−0.029) + wavelet-LLH_glcm_ClusterShade ^*^ (−0.061) + wavelet-LHH_glrlm_GrayLevelVariance ^*^ 0.061 + wavelet-LHH_glrlm_LowGrayLevelRunEmphasis ^*^ (−0.093) + wavelet-HHL_firstorder_Skewness ^*^ (−0.121) + wavelet-HHL_firstorder_Minimum ^*^ (−0.055) + wavelet-HHH_glszm_SmallAreaHighGrayLevelEmphasis ^*^ 0.041 + wavelet-HHH_glszm_SmallAreaEmphasis ^*^ 0.068.
**Rad-score** = wavelet-LLH_firstorder_Skewness ^*^ (−0.287) + wavelet-LLH_glcm_ClusterShade ^*^ (−0.372) + wavelet-LHH_glrlm_GrayLevelVariance ^*^ 0.711 + wavelet-LHH_glrlm_LowGrayLevelRunEmphasis ^*^ (−0.516) + wavelet-HHL_firstorder_Skewness ^*^ (−0.640) + wavelet-HHL_firstorder_Minimum ^*^ (−0.302) + wavelet-HHH_glszm_SmallAreaHighGrayLevelEmphasis ^*^ 0.282 + wavelet-HHH_glszm_SmallAreaEmphasis ^*^ 0.343 + 0.056.

The Radiomics Quality Score (RQS) has been recently proposed for the overall assessment of the methodological quality of radiomics-based studies (15). We also calculated it to assess the performance of our prediction models.

## Results

### Clinical data analysis

Among 102 recruited patients, 46 had good prognoses and 56 had bad prognoses. In the cross-validation set, 38 patients had good prognoses and 43 had bad prognoses. The baseline characteristics of all patients are summarized in Table 1. In the cross-validation set, the admission NIHSS score, hemoglobin levels, NLR, and D-dimer of patients with good prognoses were significantly different from those of patients with bad prognoses (*P* < 0.05). The NIHSS score at discharge (*P* < 0.05), history of hypertension (*P* < 0.1), neutrophils (*P* < 0.1), lymphocytes (*P* < 0.1), and sICH (*P* < 0.1) were also considered significant clinical factors.

**Table 1:** Correlation analysis of clinical characteristics and 3-month clinical outcome of patients in the cross-validation set and test set.

**Clinical Characteristics & imaging characteristics of manual measurement**	**Cross-validation set**			**Test set**			**Cross-validation & test set**
	**mRS (0–2)**	**mRS (3–6)**	***P***	**mRS (0–2)**	**mRS (3–6)**	***P***	***P***
No. of patients	38	43	–	8	13	–	
Age, y, median (IQR)	67 (59–71)	66 (59–70)	0.82^a^	54.5 (32–71.5)	61 (55.5–76)	0.21^a^	0.28^a^
Gender, Male	21 (55.3%)	31 (72.1%)	0.17^b^	3 (37.5%)	9 (69.2%)	0.33^b^	0.73^b^
Onset to admission (min), (median) (IQR)	198 (81.50–321.25)	160 (105–293)	0.74^a^	219 (101.25–582.75)	240 (117.5–410)	0.94^a^	0.26^a^
Onset to NCCT (min), (median) (IQR)	228 (136.25–360.00)	177 (131–301)	0.54^a^	231 (114.25–589.25)	276 (146.5–530.5)	0.80^a^	0.28^a^
Admission NIHSS, (median) (IQR)	12 (9–15)	15 (13–18)	**< 0.001** ^a^	14 (10.25–15.75)	13 (11–15)	0.44^a^	0.68^a^
The density of MCA affected side (HU), mean ± STD	63.55 ± 8.661	62.12 ± 8.71	0.46	61.50 ± 11.83	66.46 ± 11.29	0.35	0.44
The density of MCA contralateral side (HU), mean ± STD	43.32 ± 5.83	46 ± 7.77	0.84	44.88 ± 9.78	46.62 ± 5.28	0.72	0.91
Difference value of bilateral MCA (HU), mean ± STD	17.24 ± 7.09	16.12 ± 6.02	0.44	16.63 ± 6.32	19.85 ± 8.56	0.37	0.24
Density ratio of bilateral MCA, mean ± STD	1.38 ± 0.17	1.37 ± 0.16	0.69	1.39 ± 0.22	1.42 ± 0.18	0.47	0.34
Length of clot, (median) (IQR)	23.24 (14.21–29.88)	18.67 (11.1–29.81)	0.43^a^	14.89 (6.25–29.93)	16.71 (10.50–38.1)	0.45^a^	0.33^a^
Hypertension	24 (63.2%)	18 (41.9%)	**0.09** ^b^	6 (75%)	7 (53.8%)	0.61^b^	0.56^b^
Diabetes	7 (18.4%)	12 (27.9%)	0.46^b^	1 (12.5%)	3 (23.1%)	0.99^b^	0.89^b^
Arterial fibrillation	12 (31.6%)	9 (20.9%)	0.40^b^	1 (12.5%)	4 (30.8%)	0.67^b^	0.93^b^
Coronary heart disease	4 (10.5%)	9 (20.9%)	0.33^b^	2 (25%)	0 (0%)	0.26^b^	0.68^b^
Smoke	17 (44.7%)	22 (51.2%)	0.72^b^	4 (50%)	7 (53.8%)	0.78^b^	0.92^b^
Drink	13 (34.2%)	19 (44.2%)	0.49^b^	1 (12.5%)	6 (46.2%)	0.27^b^	0.79^b^
History of stroke	6 (15.8%)	10 (23.3%)	0.57^b^	1 (12.5%)	1 (7.7%)	0.69^b^	0.44^b^
Hemoglobin, (median) (IQR)	132.0 (124.0–140.2)	143.0 (129.0–149.0)	**0.02**	128.5 (103.75–149.0)	134 (123.5–140.0)	0.75	0.27^a^
WBC, (median) (IQR)	9.04 (7.29–10.82)	9.53 (7.59–11.98)	0.20	10.09 (8.59–12.94)	8.45 (7.71–11.36)	0.49	0.48^a^
Neutrophils, (median) (IQR)	7.10 (5.32–8.23)	7.88 (5.94–10.37)	**0.08**	8.78 (6.69–10.46)	7.44 (6.15–9.19)	0.49	0.33^a^
Lymphocyte, (median) (IQR)	1.44 (1.10–1.71)	1.20 (0.89–1.53)	**0.06** ^a^	1.86 (0.65–2.39)	1.09 (0.77–1.46)	0.31^a^	0.73^a^
Platelet, mean ± STD	196.16 ± 42.89	197.79 ± 53.21	0.88	172.38 ± 50.89	207.15 ± 45.24	0.12	0.79
NLR, (median) (IQR)	4.78 (3.45–6.55)	6.36 (4.61–9.42)	**0.01** ^a^	5.26(3.92–19.17)	7.32 (5.09–9.62)	0.45^a^	0.25^a^
C-reactive protein, (median) (IQR)	5.81 (3.02–8.01)	6.08 (3.02–18.30)	0.47^a^	3.07 (3.02–8.11)	7.29 (3.05–35.60)	0.25^a^	0.67^a^
Fibrinogen, mean ± STD	2.84 ± 0.79	3.11 ± 1.034	0.18	2.98 ± 0.91	3.06 ± 1.28	0.87	0.85
D-dimer, (median) (IQR)	0.96 (0.59–2.24)	2.60 (0.92–190)	**0.01** ^a^	1.00 (0.75–2.52)	0.59 (0.55–7.54)	0.46^a^	0.15^a^
Cholesterol, mean ± STD	4.18 ± 0.87	4.37 ± 1.08	0.38	4.21 ± 1.07	4.46 ± 0.91	0.57	0.73
Triglyceride, (median) (IQR)	1.60 (0.99–2.05)	1.66 (1.16–2.39)	0.28^a^	1.83 (1.05–3.21)	1.4 (0.76–1.70)	0.17^a^	0.51^a^
Onset to puncture (min), (median) (IQR)	299 (195–459.75)	301 (230–400)	0.99^a^	297.5 (203.75–662.5)	368 (235.5–690)	0.64^a^	0.36^a^
Puncture to recanalization (min)	77.50 (52.25–119)	85 (65–110)	0.20^a^	65 (51.25 ± 105)	75 (70–137.5)	0.11^a^	0.97^a^
Onset to recanalization (min)	377.5 (297.50–518.25)	390 (320–505)	0.69^a^	360 (260–747.5)	430 (368–835)	0.37^a^	0.41^a^
NIHSS at discharge, (median) (IQR)	3 (1–7)	12 (7–16)	**< 0.001** ^a^	2 (0–5)	11 (8–13.5)	**< 0.001** ^a^	1^a^
mTICI (2B and 3)	37 (97.4%)	38 (88.4%)	0.26^b^	7 (87.5%)	11 (84.6%)	0.65^b^	0.58^b^
sICH	2 (5.3%)	9 (20.9%)	**0.08** ^b^	0 (0%)	4 (30.8%)	0.24^b^	0.78^b^

Normal distribution: Mean ± standard deviation; Abnormal distribution: Median (25% Percentile−75% Percentile).

a Non–parameter test (Mann–Whitney *U*-test).

b Chi^2^ test; others: *t*-est.

The *P*-values in the last column were acquired between the whole cross-validation set and the whole test set after statistical analysis. NIHSS, National Institute of Health Stroke; WBC, white blood cells; NLR, neutrophil-to-lymphocyte ratio; mTICI, modified thrombolysis in cerebral infarction; sICH, symptomatic intracerebral hemorrhage.

In the cross-validation set, 92.6% (75/81) of the patients achieved successful reperfusion (mTICI 2b/3). The successful reperfusion rate between patients with a good prognosis and those with a bad prognosis was not significantly different (97.4 vs. 88.4%, *P* = 0.26). The HMCAS was not significantly correlated with the 90-day prognosis regardless of the density and length of the clot. In addition, there were no significant differences in age, sex, time from onset to puncture, procedure time, and time from onset to reperfusion between patients with a good prognosis and those with a bad prognosis (*P* > 0.05). In the test set, only the NIHSS score at discharge showed a strong correlation with the bad prognosis. No significant correlations were found between the prognosis and other risk factors. In addition, the *p*-values of all variables between the cross-validation set and the test set were more than 0.05, indicating the data of the two sets are evenly distributed (Table 1).

### Extraction and selection of NCCT semantic features and RFs

The coefficients of 8 RFs are shown in Supplementary Table 1. The classification performance of the fusion RF (Lasso-score) with the feature coefficient value analyzed by the LASSO algorithm between the two groups in both the cross-validation and test sets is shown in Supplementary Figure 3, Table 1.

### Development of prediction models

Model 1 (CM pre-MT) was established with 7 preoperative CFs with a *p*-value of < 0.1 before the operation, and the coefficients of the features are shown in Supplementary Figure 4. The ROCs of the training and verification sets were plotted using the model with optimal parameters from the 5-fold cross-validation. In CM pre-MT, the mean areas under the curve (AUC) (95%CI) of the training set was 0.76 ± 0.03 (0.71–0.81); the mean AUC (95%CI) of the validation set was 0.67 ± 0.13 (0.55–0.79); AUC (95%CI) of the cross-validation set was 0.741 (0.634–0.848); AUC (95%CI) of the test set was 0.596 (0.312–0.881).

Model 2 (RM) predicts poor prognosis with 8 RFs, whose coefficients are shown in Supplementary Figure 5. In RM, the mean AUC (95%CI) of the training set and the validation set was 0.84 ± 0.04 (0.80–0.88) and 0.78 ± 0.15 (0.64–0.85), respectively; AUC (95%CI) of the cross-validation set and the test set was 0.829 (0.741–0.918) and 0.798 (0.598–0.998), respectively.

**Figure 4:**
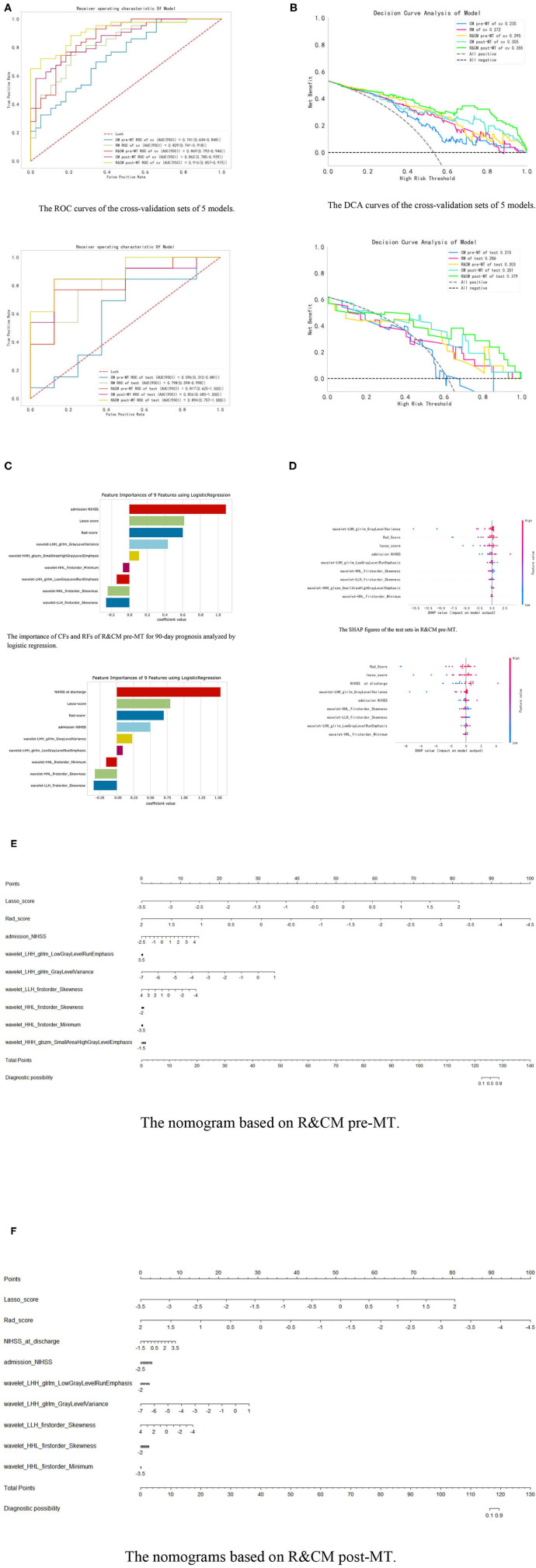
Integration of related results of five models. **(A)** The ROC curves of five models in cross-validation set and test set, respectively. **(B)** The DCA curves of the five models in the test set. When the high-risk threshold is greater than 0.5, 4 DCA curves are higher than positive curves, indicating that the four models have good returns. Moreover, the AUC of the R&CM post-MT (0.353) was larger than that of the R&CM pre-MT (0.296), indicating that the R&CM post-MT had better prediction performance. **(C)** The coefficient of CFs and RFs for 90-day prognosis in R&CM pre-MT and R&CM post-MT, respectively. **(D)** The SHAP figures of the test sets in R&CM pre-MT and R&CM post-MT, respectively. Each dot stands for a patient with a feature value from low to high, showing as a color from blue to red. The SHAP value indicates the contribution of the features to the model. The nomograms based on the **(E)** R&CM pre-MT and **(F)** R&CM post-MT. The nomogram critical score before MT was 125.5 and the nomogram critical score after MT was 117.9, indicating that the probability of bad prognosis of patients with HMCAS on NCCT is >50% when the total score is >125.5 or 117.9. The higher the score is, the higher the probability of a bad prognosis after MT.

Model 3 (R&CM pre-MT) was established using preoperative CFs and RFs after recursive feature elimination (RFE). The admission NIHSS (*P* < 0.001) and 8 RFs were included in this model whose importance and coefficients of the features are shown in Figure 4C. In R&CM pre-MT, the mean AUC (95%CI) of the training set and the validation set was 0.88 ± 0.02 (0.85–0.92) and 0.79 ± 0.12 (0.67–0.87), respectively; AUC (95%CI) of the cross-validation set and the test set was 0.869 (0.792–0.946) and 0.817 (0.625–1.000), respectively.

Model 4 (CM post-MT) predicts poor prognosis with post-operative CFs, which include four preoperative CFs with a *p*-value of < 0.05 (admission NIHSS, hemoglobin levels, NLR, and D-dimer) and the NIHSS at discharge. The coefficients of the features are shown in Supplementary Figure 6. The mean AUC (95%CI) of the training set and the validation set was 0.86 ± 0.01 (0.83–0.90) and 0.83 ± 0.06 (0.73–0.91), respectively; AUC (95%CI) of the cross-validation set and the test set was 0.862 (0.785–0.939) and 0.856 (0.685–1.000), respectively.

Model 5 (R&CM post-MT) was established using post-operative CFs and RFs after RFE. The importance and coefficients of the features are shown in Figure 4C. The mean AUC (95%CI) of the training set and the validation set was 0.93 ± 0.01 (0.91–0.96) and 0.87 ± 0.09 (0.80–0.95), respectively; AUC (95%CI) of the cross-validation set and the test set was 0.916 (0.857–0.975) and 0.894 (0.754–1.000), respectively. The comparison of ROC curves of five models on the cross-validation and test sets is shown in Figure 4A.

Among the five models, the R&CM post-MT (0.894 [95%CI, 0.757–1.000]) showed the best discriminative performance. The second is the CM post-MT (0.856 [95% CI 0.685–1.000]), which had better discriminative performance than the other 3 models established before MT. In addition, the R&CM pre-MT (0.817 [95% CI 0.625–1.000]) also shows better discriminative performance than the RM (0.798 [95% CI 0.598–0.998]). The decision curve analyses (DCAs) of the five models on the cross-validation and test sets are shown in Figure 4B. Figure 4D shows the distribution of each feature with the Shapley values for each patient of the R&CM pre-MT and R&CM post-MT in descending order. Lasso-score and Rad-score have higher Shapley values of all features in both confusion models. The nomograms of R&CM pre-MT and R&CM post-MT were built to visualize the weight coefficient of each feature (Figures 4E, F). The sensitivity, specificity, and other parameters of all prediction models are shown in Table 2.

**Table 2:** The different evaluation parameters in five models.

		**AUC**	**AUC std**	**SEN**	**SEN std**	**SPE**	**SPE std**	**Precision**	**Precision std**	**Recall**	**Recallstd**	**F1-Score**	**F1-Score std**	**Acc**	**Acc std**
CM pre-MT	The training set of cross-validation	0.76	0.03	0.77	0.03	0.60	0.03	0.69	0.01	0.77	0.03	0.73	0.02	0.69	0.01
	The validation set of cross-validation	0.67	0.13	0.69	0.11	0.55	0.08	0.63	0.07	0.69	0.11	0.66	0.08	0.63	0.07
	Cross-validation set	0.741	0	0.77	0	0.55	0	0.66	0	0.77	0	0.71	0	0.67	0
	Test set	0.596	0	0.62	0	0.63	0	0.73	0	0.62	0	0.67	0	0.62	0
RM	The training set of cross-validation	0.84	0.04	0.81	0.04	0.69	0.05	0.75	0.05	0.81	0.04	0.78	0.04	0.75	0.05
	The validation set of cross-validation	0.78	0.15	0.71	0.18	0.61	0.23	0.69	0.17	0.71	0.18	0.69	0.15	0.67	0.17
	Cross-validation set	0.829	0	0.79	0	0.71	0	0.76	0	0.79	0	0.77	0	0.75	0
	Test set	0.798	0	0.54	0	0.88	0	0.88	0	0.54	0	0.67	0	0.67	0
R&CM pre-MT	The training set of cross-validation	0.88	0.02	0.82	0.04	0.76	0.03	0.79	0.02	0.82	0.04	0.81	0.03	0.79	0.03
	The validation set of cross-validation	0.79	0.12	0.64	0.19	0.64	0.16	0.67	0.11	0.64	0.19	0.65	0.11	0.64	0.10
	Cross-validation set	0.869	0	0.84	0	0.74	0	0.78	0	0.84	0	0.81	0	0.79	0
	Test set	0.817	0	0.69	0	0.88	0	0.90	0	0.69	0	0.78	0	0.76	0
CM post-MT	The training set of cross-validation	0.86	0.01	0.76	0.04	0.79	0.03	0.80	0	0.76	0.04	0.78	0.02	0.77	0.02
	The validation set of cross-validation	0.83	0.06	0.73	0.11	0.77	0.14	0.79	0.08	0.73	0.11	0.75	0.05	0.74	0.05
	Cross-validation set	0.862	0	0.77	0	0.79	0	0.80	0	0.77	0	0.79	0	0.78	0
	Test set	0.856	0	0.77	0	0.88	0	0.91	0	0.77	0	0.83	0	0.81	0
R&CM post-MT	The training set of cross-validation	0.93	0.01	0.87	0.03	0.82	0.02	0.85	0.02	0.87	0.03	0.86	0.02	0.85	0.02
	The validation set of cross-validation	0.87	0.09	0.79	0.16	0.77	0.17	0.81	0.13	0.79	0.16	0.79	0.10	0.78	0.10
	Cross-validation set	0.916	0	0.86	0	0.82	0	0.84	0	0.86	0	0.85	0	0.84	0
	Test set	0.894	0	0.69	0	0.88	0	0.90	0	0.69	0	0.78	0	0.76	0

The results of the DeLong test revealed that the *p*-value between the R&CM pre-MT and R&CM post-MT is 0.07. In our study, the RQS score was 12 (maximum score = 36), corresponding to 33.3%; further details are provided in Supplementary Table 3. The results in predicting the long-term prognosis after MT are encouraging.

## Discussion

This is the first study to explore the prognostic value of the HMCAS on NCCT combined with radionics among patients undergoing MT. The results indicate that radiomics-based HMCAS has a limited ability to predict poor prognosis, so CFs should be included when establishing prediction modes. In this study, we first used preoperative features to establish prediction models to observe their performance in predicting prognosis. While considering that treatment-related factors also play an important role in the prognosis (16), we also add post-operative features to establish prediction models. On the one hand, the model with post-operative features could predict patients' prognoses more accurately when applied after the operation; on the other hand, we can learn about the prediction of R&CM pre-MT in comparison with R&CM post-MT. The gap between the two is so small that the R&CM pre-MT could also be used to provide guidance on treatment strategy selection.

A few studies have explored the role of thrombus in the endovascular treatment of AIS (9), but most of them did not consider RFs. Kim et al. showed that there was no significant correlation between HMCAS and treatment outcomes after MT (5). Our data showed that the density, density ratio of the affected side to the contralateral side, and length of the clot were not correlated with the prognosis of patients after MT, which was in line with the study by Alejandro et al. (12).

In both R&CM pre-MT and R&CM post-MT, the NIHSS score at admission contributed greatly to the efficiency of the model, which indicates a strong correlation between early neurological deterioration and clinical functional outcome, which is consistent with the study of Thomas *et al* in the era of pre-thrombectomy (17).

Our data showed that the long-term prognosis of patients after MT may be affected by clot-related RFs, suggesting that these RFs may be used to guide treatment selection. In the RM, the red color seems to dominate a big share in the visual red blueprint for the texture feature “wavelet-LHH_glrlm_GrayLevelVariance,” the representative texture feature with the maximum correlation; the blue color seems to dominate a big share for the texture feature “wavelet-HHH_glszm_SmallAreaHighGrayLevelEmphasis,” the representative texture feature with the minimum correlation. We infer that long-term prognosis may be reflected by the color composition ratio of the RFs. In addition, we could see that the color composition of all RFs is more complex in the poor prognosis group compared to the good prognosis group; we speculated that patients with heterogeneous thrombus are more likely to have a bad prognosis. These findings were contradictory to the study conducted by Jeremy et al. (9), probably because the endpoint of their study was recanalization after MT, while ours was a 90-day prognosis. The study conducted by T. R. Patel *et al*. showed that clots with the ordered structure were easier to remove (7), which seems to be echoed by our conclusion. However, the underlying laws between RFs and histopathology remain to be explored.

A recent study conducted by Lin *et al*. showed that the NLR of > 3.5 improves the prediction performance of the HAS on NCCT for the short-term clinical outcome (the mRS score at discharge) (18). Our data showed that the NLR was correlated with long-term clinical outcomes, indicating that the development of stroke may be related to inflammation.

Our study has several limitations. First, this is a single-center study with a small sample size and lacks further verification. Second, different endovascular strategies and variations in the parameters of different MT devices may reduce the repeatability of the experiment. While stent retriever and direct thromboaspiration are the MT strategies used in our study, they are proven to be comparable in terms of overall effectiveness (19, 20). Third, patients without a hyperdense vessel sign in MCA were not included in this study because it was difficult to identify the scope of the vessel affected when outlining the ROI. In-depth learning may be needed in future investigations to solve this problem. Finally, given the multimodal CT examinations are still not available in most of the basic-level hospitals, we did not assess the collateral circulation state in order to get more clues from the NCCT and make the established models to benefit more patients.

In conclusion, our study revealed that radiomics-based HMCAS on admission NCCT before MT may be not only a potential imaging biomarker for treatment selection but also may guide the functional recovery of patients with AIS after MT. The prediction model should be established with both RFs and CFs.

## Data availability statement

The original contributions presented in the study are included in the article/Supplementary material, further inquiries can be directed to the corresponding author.

## Ethics statement

The studies involving human participants were reviewed and approved by Medical Ethics Committee of the First Hospital of Jilin University. The patients/participants provided their written informed consent to participate in this study. Written informed consent was obtained from the individual(s) for the publication of any potentially identifiable images or data included in this article.

## Author contributions

LL and ML conceived the study. LL was involved in data collection, study design, data analysis, and manuscript writing. ML contributed to building models, data analysis, manuscript writing, and revision. ZC and FL were mainly responsible for data collection. MZ also played an important role in data analysis and building models. DT and HZ supervised the study and revised the manuscript. All authors contributed to the article and approved the submitted version.
