# Supplementary material for: Prognostic value of radiomics-based hyperdense middle cerebral artery sign for patients with acute ischemic stroke after thrombectomy strategy
Source: Front Neurol. 2023 Jan 13;13:1037204. doi: 10.3389/fneur.2022.1037204 (PMC9880054; doi:10.3389/fneur.2022.1037204)
Supplement: Supplementary file 1 [file Data_Sheet_1.pdf]

Supplementary Material

Prognostic value of Radiomics-Based Hyperdense Middle Cerebral Artery Sign for Patients with Acute Ischemic Stroke after Thrombectomy Strategy

Linna Li [1], Mingyang Li [1], Zhongping Chen [1], Fei Lu[1], Min Zhao[2], Huimao Zhang[1], Dan Tong[1]*

[1] Department of Radiology, The First Hospital of Jilin University, Changchun, Jilin, 130021, China

[2] Pharmaceutical Diagnostics, GE Healthcare, Beijing, 100176, China

* **Correspondence:** Dan Tong   Email :tongdan@jlu.edu.cn

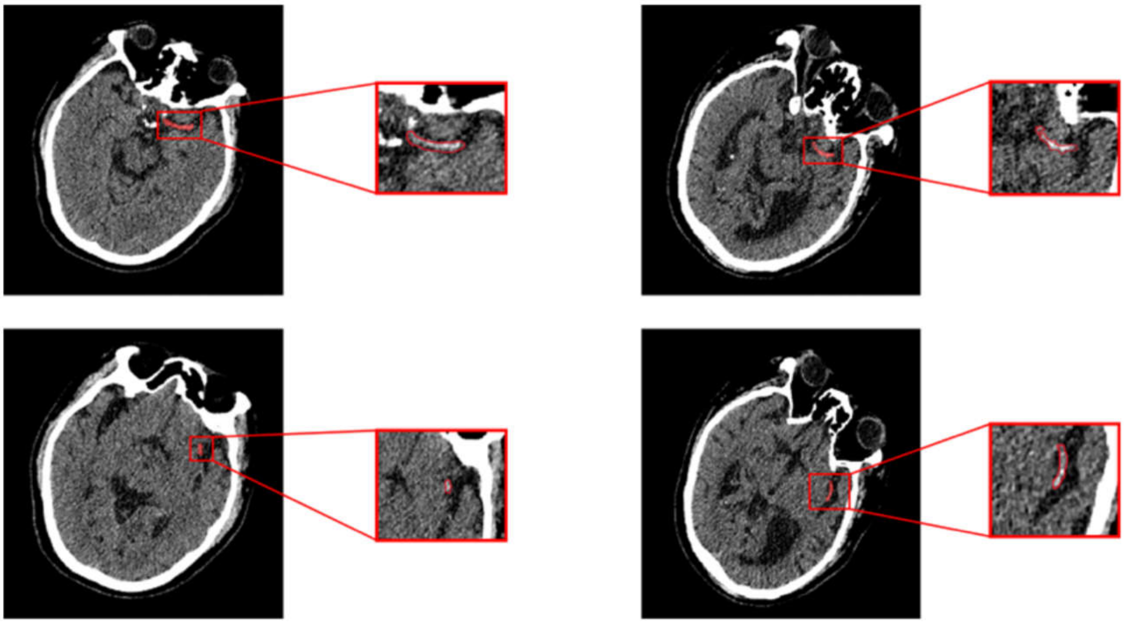

A   B

Supplementary Figure 1. (A) An example of patient with a good prognosis. (B) An example of the patient with a bad prognosis.

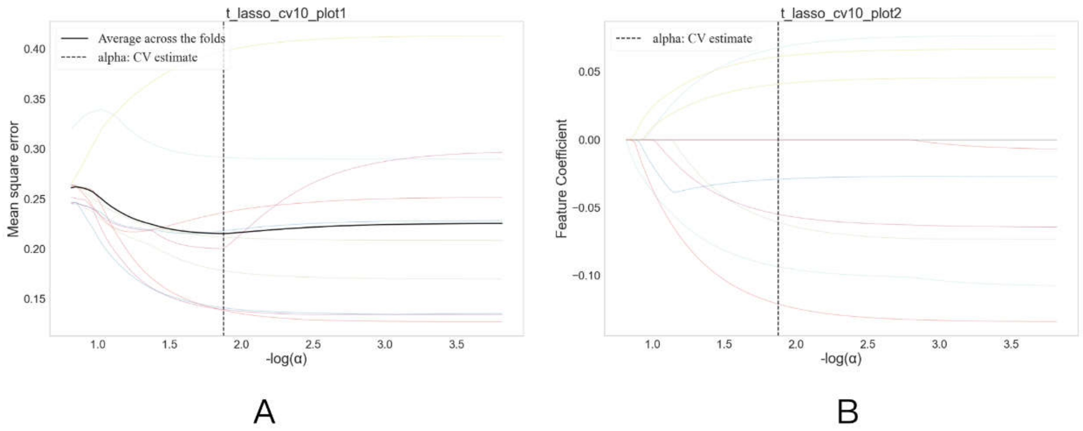

Supplementary Figure 2. (A) The relationship between the mean square of the LASSO 10-fold cross-validation and the value of -log(α). The thick solid line is the mean LASSO 10-fold cross-validation curve and the dotted line is the LASSO regularized curve. (B) The relationship between the feature coefficient and the value of -log(α). The thin dotted line is the feature coefficient value of each fold at different -log(α) values. The thick dotted line is the value of -log(α) at the minimum mean square error.

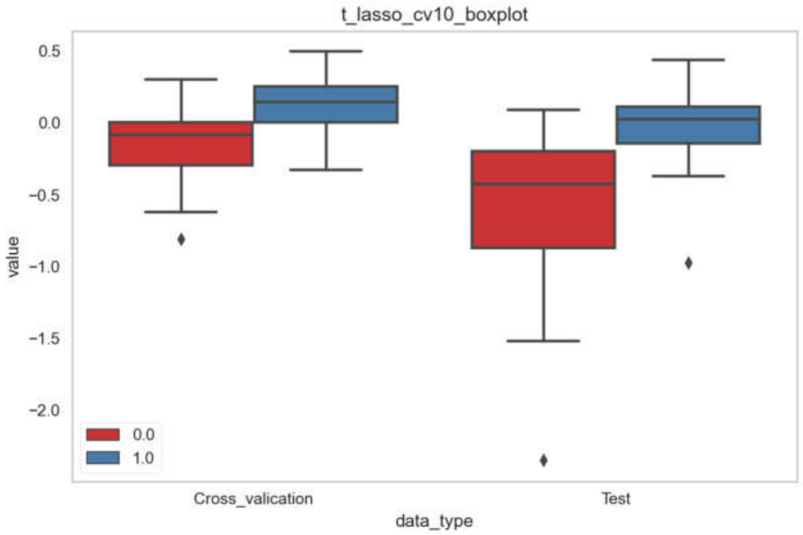

Supplementary Figure 3. Box plot of the fusion RF (named Lasso-score) on the cross-validation and test sets. A label of 0 indicates good prognosis, while a label of 1 indicates bad prognosis.

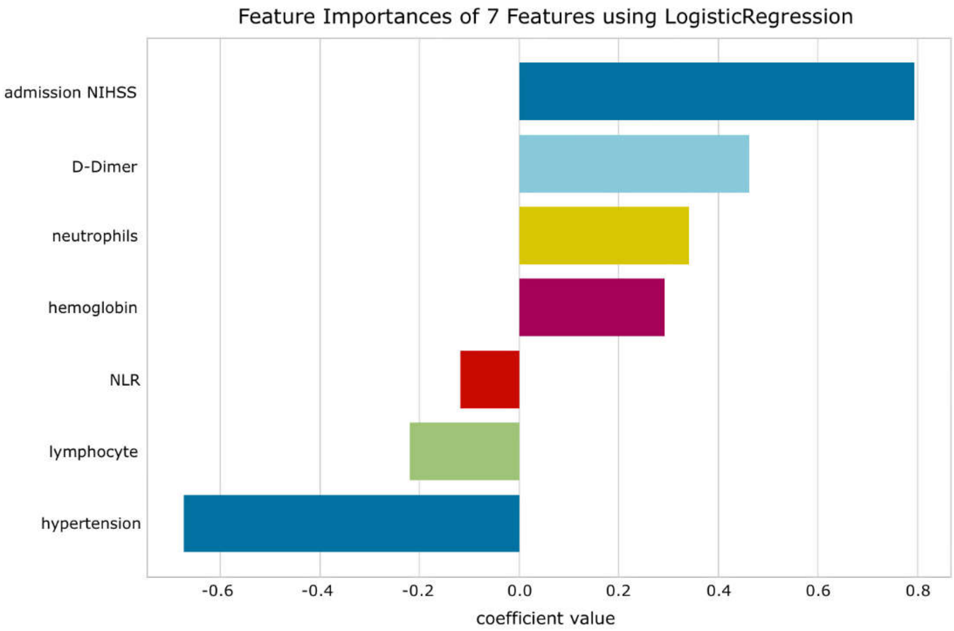

Supplementary Figure 4. The importance of CFs pre-MT for 90-day prognosis analyzed by logistic regression.

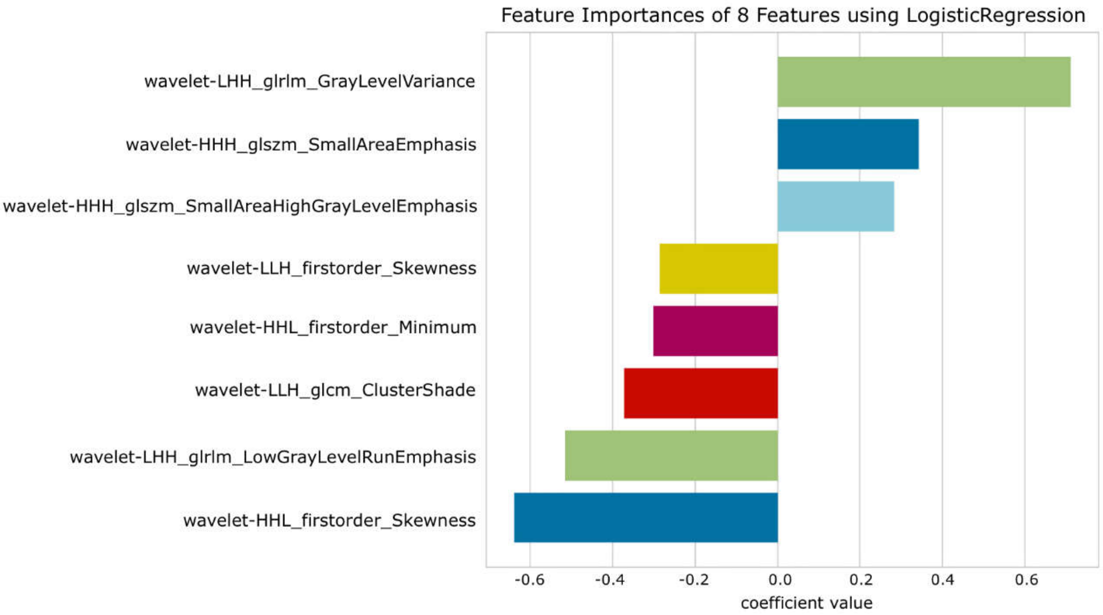

Supplementary Figure 5. The importance of RFs for 90-day prognosis analyzed by logistic regression.

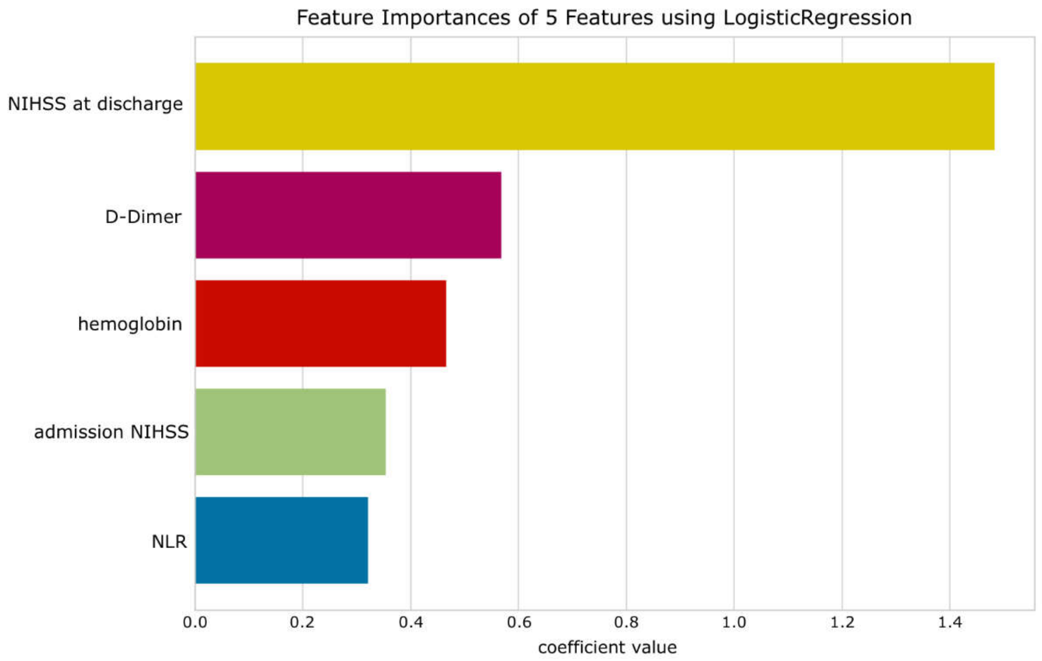

Supplementary Figure 6. The importance of CFs post-MT for 90-day prognosis analyzed by logistic regression.

Supplementary Table 1. Coefficients of RFs analyzed by the LASSO algorithm

**Name of RFs**	**Coefficient**
wavelet-LLH_firstorder_Skewness	-0.029
wavelet-LLH_glcm_ClusterShade	-0.061
wavelet-LHH_glrlm_GrayLevelVariance	0.061
wavelet-LHH_glrlm_LowGrayLevelRunEmphasis	-0.093
wavelet-HHL_firstorder_Skewness	-0.121
wavelet-HHL_firstorder_Minimum	-0.055
wavelet-HHH_glszm_SmallAreaHighGrayLevelEmphasis	0.041

wavelet-HHH_glszm_SmallAreaEmphasis	0.068

wavelet: wavelet transform; LLH, LHH, HHL and HHH: subbands of the wavelet transform; firstorder: first-order feature; glcm: gray-level co-occurrence matrix; glrlm: gray-level run-length matrix; glszm: gray-level size-zone matrix.

Supplementary Table 2. Intra-observer and inter-observer reproducibility of simple imaging features

	Intraclass Correlation Coefficient (95% CI)	
	Intra-observer	Inter-observer
The density of MCA affected side	0.924(0.737-0.967)	0.837(0.767-0.886)
The density of MCA contralateral side	0.903(0.715-0.955)	0.858(0.745-0.915)
Difference value of bilateral MCA	0.931(0.899-0.953)	0.924(0.889-0.948)
Density ratio of bilateral MCA	0.913(0.874-0.940)	0.888(0.836-0.924)
Length of clot	0.819(0.739-0.876)	0.923(0.887-0.948)

CI: Confidence interval; MCA: middle cerebral artery

Supplementary Table 3. RQS score: Our experiment met criteria 1(+1), 2(+1), 5(+3), 6(+1), 9(+1), 10(+1), 12(+2), 14(+2); therefore, the total score was 12.

**Criteria**		**Points**
**1**	**Image protocol quality-well-documented image protocols (for example, contrast, slice thickness, energy, etc.) and/or usage of public image protocols allow reproducibility/replicability**	**+1 (if protocols are well-documented) +1 (if public protocol is used)**

**2**	**Multiple segmentations-possible actions are: segmentation by different physicians/algorithms/software, perturbing segmentations by (random) noise, segmentation at different breathing cycles. Analyse feature robustness to segmentation variabilities**	**+1**
3	Phantom study on all scanners-detect inter-scanner differences and vendor-dependent features. Analyse feature robustness to these sources of variability	+1
4	Imaging at multiple time points-collect images of individuals at additional time points. Analyse feature robustness to temporal variabilities (for example, organ movement, organ expansion/ shrinkage)	+1
**5**	**Feature reduction or adjustment for multiple testing-decreases the risk of overfitting. Overfitting is inevitable if the number of features exceeds the number of samples. Consider feature robustness when selecting features**	**-3(if neither measure is implemented) +3(if either measure is implemented)**
**6**	**Multivariable analysis with non radiomics features (for example, EGFR mutation) is expected to provide a more holistic model. Permits correlating/inferencing between radiomics and non radiomics features**	**+1**
7	Detect and discuss biological correlates-demonstration of phenotypic differences (possibly associated with underlying gene-protein expression patterns) deepens understanding of radiomics and biology	+1
8	Cut-off analyses-determine risk groups by either the median, a previously published cut-off or report a continuous risk variable Reduces the risk of reporting overly optimistic results	+1

**9**	**Discrimination statistics-report discrimination statistics (for example, C-statistic, ROC curve, AUC) and their statistical significance (for example, p-values, confidence intervals). One can also apply resampling method (for example, bootstrapping, cross-validation)**	**+1 (if a discrimination statistic and its statistical significance are reported) +1(if a resampling method technique is also applied)**
**10**	**Calibration statistics-report calibration statistics (for example, Calibration-in-the-large/slope, calibration plots) and their statistical significance (for example, P-values, confidence intervals). One can also apply resampling method (for example, bootstrapping. cross-validation)**	**+1 (if a calibration statistic and its statistical significance are reported) +1 (if a resampling method technique is also applied)**
11	Prospective study registered in a trial database-provides the highest level of evidence supporting the clinical validity and usefulness of the radiomics biomarker	+7 (for prospective validation of a radiomics signature in an appropriate trial)
**12**	**Validation-the validation is performed without retraining and without adaptation of the cut-off value, provides crucial information with regard to credible clinical performance**	**-5(if validation is missing) +2(if validation is based on a dataset from the same institute) +3(if validation is based on a dataset from another institute) +4(if validation is based on two datasets from two distinct institutes) +4(if the study validate is a previously published signature) +5(if validation is based on three or more datasets from distinct**

		**institutes)**<br><br>***Datasets should be of comparable size and should have at least 10events per model feature**
13	Comparison to 'gold standard' -assess the extent to which the model agrees with/is superior to the current 'gold standard' method (for example, TNM-staging for survival prediction). This comparison shows the added value of radiomics	+2
**14**	**Potential clinical utility-report on the current and potential application of the model in a clinical setting (for example, decision curve analysis).**	**+2**
15	Cost-effectiveness analysis-report on the cost-effectiveness of the clinical application (for example, QALYs generated)	+1
16	Open science and data-make code and data publicly available. Open science facilitates knowledge transfer and reproducibility of the study	1(if scans are open source) +1(if region of interest segmentations are open source) +1(if code is open source) +1(if radiomics features are calculated on a set of representative ROIs and the calculated features and representative ROIs are open source)
**Total points (36=100%)**		
